# Developmental perspectives on HiTOP psychosis superspectrum: Unveiling pitfalls and theoretical fallacies

**DOI:** 10.3389/fpsyt.2025.1523025

**Published:** 2025-05-16

**Authors:** Michele Poletti, Antonio Preti, Andrea Raballo

**Affiliations:** ^1^ Department of Mental Health and Pathological Addiction, Child and Adolescent Neuropsychiatry Service, Azienda USL-IRCCS di Reggio Emilia, Reggio Emilia, Italy; ^2^ Department of Neuroscience, University of Turin, Turin, Italy; ^3^ Chair of Psychiatry, Faculty of Biomedical Sciences, University of Southern Switzerland, Lugano, Switzerland; ^4^ Cantonal Sociopsychiatric Organisation, Mendrisio, Switzerland

**Keywords:** psychosis, HiTOP classification, psychoticism, detachment, cognitive develoipment, clinical high-risk for psychosis, at-risk mental state

## Introduction

The Hierarchical Taxonomy of Psychopathology (HiTOP) consortium recently defined the psychosis superspectrum (1) and focused on its nosology, etiology, and lifespan development (2). In the HiTOP bottom-up conceptual model, the psychosis superspectrum comprises two spectra - psychoticism and detachment - each consisting of traits and symptoms.

*Psychoticism* includes four traits (fantasy proneness, unusual experiences, unusual beliefs, and peculiarity), two core symptom components (reality distortion and disorganization), and two additional symptom components (mania and dissociation), which are provisionally included pending further investigation (3).

*Detachment* is composed of four traits (emotional detachment, anhedonia, social withdrawal, and romantic disinterest) and two symptom components (inexpressivity and avolition).

The HiTOP model of psychosis is not without critical points, particularly regarding the two-spectrum structure of the superspectrum (4); for example, a meta-analysis found that detachment was consistent with general psychopathology rather than with the negative dimension of psychosis (4, 5) and in a recent developmentally-informed HiTOP model on a symptom-based, large-scale study with youth (6), psychotic symptoms were included in the externalizing spectrum rather than each forming separate factors.

Acknowledging these limitations (4–6) - which warrant further empirical investigation and model refinement (7) - this contribution focuses on the dimensional nuances of the psychosis superspectrum as articulated by the HiTOP consortium (1), outlining a possible longitudinal development of psychosis across developmental and clinical stages (2). This developmental perspective presents potential shortcomings and misconceptions that require further examination to guide future empirical research, inspired by the HiTOP model, on psychosis.

### Developmental features of psychoticism

A first oversimplification may lie in the overall conceptualization of the developmental trajectory leading to the emergence of psychoticism, i.e. its ontogenesis. HiTOP authors (2) stated that individual differences in psychoticism are apparent by middle school, citing a study on childhood psychosis (8). However, it is well known that even childhood-onset schizophrenia does not begin with the early (or very early) appearance of diagnostic psychotic symptoms (9). Therefore, the low prevalent condition of childhood psychosis may not be the optimal model for tracing the earliest emergence of individual differences in psychoticism. Similarly, the moderate rank-order stability stated for traits of psychoticism from ages 7 to 12 (2) is based on a longitudinal study of the offspring of individuals with schizophrenia or bipolar disorder (10). Familial high-risk vulnerability is a questionable model for generalizing the hypothesis of developmental stability in psychoticism to the broader general population, which, by definition, is presumably not at familial-genetic risk (11).

These examples illustrate a tendency toward oversimplification in describing the development of the psychotic superspectrum, i.e. its ontogenetic unfolding along developmental stages. This is particularly evident in the conflation of more stable features - putative traits of psychoticism - with more transient features - symptoms of psychoticism, such as psychotic-like experiences. In contrast, a more realistic and clinically faithful developmental perspective suggests that vulnerability traits for psychoticism may already emerge in childhood as a consequence of neurobiological constraints related to genetic and/or environmental risk factors (11, 12). These traits may phenotypically manifest as, for example, childhood oddity or intersubjective difficulties with peers, which could later evolve and structure into avoidant or cluster A-like (paranoid, schizotypal, schizoid) personality traits, laying the groundwork for the eventual emergence of reality-distortion symptoms (13).

In this regard, also the statement that the Clinical High-Risk for Psychosis (CHR-P) stage serves as a bridging link - or intermediate stage - between childhood subclinical manifestations of psychoticism and full-blown reality distortion (hallucinations and delusions) in early adulthood (2) represents another oversimplification, despite its apparent conceptual linearity. As highlighted in the clinical staging model, the prodromal stage indexed by CHR-P is characterized by the first appearance of psychotic manifestations in the form of attenuated symptoms (14). In contrast, childhood premorbid stages may only involve endophenotypic features that are relatively nonspecific as prognostic precursors of psychosis (12).

Contemporary conceptualizations of psychosis, shaped by its prevailing reduction to positive symptoms, could tend to overemphasize delusional-hallucinatory features in risk assessment and transition prediction. This often comes at the expense of attention to negative symptoms, assessed but poorly considered in specific instruments as CAARMS and SIPS while determining the putative psychometric transition to psychosis in CHR-P individuals (15). Only about one-third of CHR-P individuals undergo a psychometric transition to psychosis, suggesting that prognostic accuracy based solely on the symptomatic level of attenuated positive psychotic symptoms in adolescence is significantly lower than what might be achieved by also considering levels of negative symptoms (15). Therefore, the CHR-P construct could only partially fit to study prodromal or intermediates stages of psychoticism according to the HiTOP model.

Moreover, in examining the psychoticism spectrum, it is essential to distinguish traits from symptoms, as they likely differ in ontogenesis, timing of phenotypic onset, longitudinal course, neurobiological determinants, and prognostic significance (16, 17). Finally, further refinements of the HiTOP model should be more selective in the supporting evidence used and more rigorous in articulating a clinically coherent interpretation. Childhood-onset schizophrenia, familial high-risk, CHR-P, and psychotic-like experiences are not interchangeable constructs, nor are they equally central proxies for the ontogenesis of psychoticism across developmental and clinical stages.

### Developmental features of detachment

Keeping in mind the critiques regarding the loading of detachment onto the negative dimension of the psychosis superspectrum (5), a similar reasoning can be applied to the development of detachment itself, whose accurate diagnosis presents greater clinical challenges than that of psychoticism - particularly in younger individuals.

Recognizing the early roots of detachment is more complex than for psychoticism. Detachment may originate from early disruptions in intersubjective attunement with peers, beginning in childhood. These disruptions can later manifest as social anhedonia when peer relationships become central to identity formation during adolescence (18). Therefore, detachment typically precedes the symptomatic features of psychoticism, such as reality distortion (19). Detecting detachment before adolescence - and distinguishing it from the secondary effects of affective symptoms, which belong to the emotional dysfunction superspectrum (20) - is especially difficult and often relies on observable behaviors such as social withdrawal. In this perspective EPA guidelines on assessment of negative symptoms (21) clearly encouraged that clinical observation of social withdrawal should also focus on inexpressivity and should be accompanied by the assessment of subjective experience of social amotivation, not limited to self-report as in the HiTOP model.

### Developmental features of cognition

Although the shortcoming of not including cognitive impairment in the HiTOP model of psychosis, it has been suggested that cognitive deficits within the psychosis superspectrum emerge more than a decade before the onset of clinically significant psychotic symptoms (2). From a neurodevelopmental perspective on the ontogenesis of psychosis, traits of psychoticism and detachment may be present from childhood but typically become more pronounced and structured during adolescence, forming the foundation for related symptoms. According to this developmental framework, cognitive impairment should be understood as a developmental lag due to neurodevelopmental constraints rather than a decline from a previously normative developmental trajectory, particularly when compared to healthy age-matched controls (22).

Thus, in individuals with a presumed transgenerational and neurobiological vulnerability (e.g., schizotaxia), nonspecific cognitive symptoms may represent the earliest phenotypic premorbid expressions (12), particularly in the neurocognitive domain of motor coordination (23–25).

## Conclusions

The HiTOP model’s depiction of the psychosis superspectrum through the constructs of psychoticism and detachment offers a compelling framework (1). If the HiTOP perspective is robust, though not yet conclusive (4–6), in capturing the structure of psychosis once it has manifested, its dynamic characterization across development - considering developmental phases (childhood, adolescence, adulthood) and clinical stages (premorbid phase, prodromal phase, clinical phase) - remains preliminary (2) requiring further refinement.

To address the ontogenesis of psychosis while adhering to the HiTOP model’s framework, particular attention must be paid to the selected populations and measurement tools used when comparing them to healthy controls. As previously noted, childhood-onset schizophrenia, familial high-risk, clinical high-risk, and psychotic-like experiences are not interchangeable constructs for assessing psychosis risk across development and clinical stages.

Finally, it is important to emphasize that while psychosis appears to be a transdiagnostic construct, the ontogenesis of the process culminating in its phenotypic manifestation is not necessarily transdiagnostic - that is, it may not be analogous across different mental disorders. For example, Self-disorders affecting the Minimal or Basic Self, in terms of anomalous subjective experiences, are specific to the schizophrenia spectrum and underlie the emergence of psychotic manifestations (26).

Concluding, to advance and deepen our understanding of developmental unfolding of psychoticism and detachment, future longitudinal studies must employ tailored instruments and include a range of pre-test risk populations.
